# Distinct Factors Secreted by Adipose Stromal Cells Protect the Endothelium From Barrier Dysfunction and Apoptosis

**DOI:** 10.3389/fcell.2020.584653

**Published:** 2020-09-30

**Authors:** Hongyan Lu, Stephanie Merfeld-Clauss, Yameena Jawed, Keith L. March, Michael E. Coleman, Natalia V. Bogatcheva

**Affiliations:** ^1^Division of Cardiology, Department of Medicine, Indiana University, Indianapolis, IN, United States; ^2^Indiana Center for Vascular Biology and Medicine and Vascular and Cardiac Adult Stem Cell Therapy Center, Indianapolis, IN, United States; ^3^Roudebush Veteran Affairs Medical Center, Indianapolis, IN, United States; ^4^Theratome Bio, Inc., Indianapolis, IN, United States; ^5^Division of Pulmonary, Sleep and Critical Care, Department of Medicine, Indiana University, Indianapolis, IN, United States

**Keywords:** ASC, conditioned media, endothelial cells, barrier dysfunction, apoptosis

## Abstract

We have shown previously that adipose stromal cell (ASC)-derived conditioned media (CM) limited lung injury, endothelial barrier dysfunction, and apoptosis. Here, we used endothelial hyperpermeability and apoptosis assays to investigate how concentration processes affect endothelium-directed bioactivity of ASC-CM and to gain information on the nature of bioactive factors. Comparison of ASC-CM concentrated with differential molecular weight (MW) cutoff filters showed that endothelial barrier protection depended on the species-specific factors in ASC-CM fractionated with MW > 50 kDa. Known barrier regulators—keratin growth factor (KGF), vascular endothelial growth factor (VEGF), and hepatocyte growth factor (HGF)—were detected in ASC-CM fraction of > 100 kDa. Pretreatment of endothelial monolayers with concentrations of KGF, VEGF, and HGF detected in ASC-CM showed that only KGF and HGF protect the endothelium from barrier dysfunction. Depletion of KGF and HGF from ASC-CM attenuated ASC-CM’s ability to protect the endothelial barrier. In contrast to barrier-protective factors, apoptosis-protective factors fractionated with MW < 3 kDa and were not species-specific. Application of donors of apoptosis-mitigating gases showed that the CO donor carbon monoxide-releasing molecule 2 (CORM2) protected the endothelium from apoptosis, while the H_2_S donor NaSH did not. Knockdown of CO-generating heme oxygenase 1 in ASC attenuated ASC-CM’s ability to protect the endothelium from apoptosis. We have shown that tumor necrosis factor alpha (TNFα)-induced apoptosis in endothelium is c-Jun N-terminal kinase (JNK)-dependent, and JNK activation is inhibited by ASC-CM pretreatment of endothelial cells. ASC-CM from heme oxygenase 1-depleted ASC displayed attenuated ability to suppress endothelial JNK activation, suggesting that CO-mediated protection of the endothelium from apoptosis is achieved by the downregulation of the JNK pathway. Altogether, our results demonstrate that the concentration of ASC-CM with low MW cutoff filters significantly reduces its anti-apoptotic activity while preserving its barrier-protective activity.

## Introduction

Adipose stromal cells (ASC) are a population of adult mesenchymal stromal (stem) cells first isolated from adipose tissue in Zuk et al. (2001). They have self-renewing properties and can differentiate into several cell lineages, but like other stromal cells, their therapeutic potential is thought to be associated with the secretion of protective and regenerative factors rather than engraftment and trans-differentiation (Liang et al., 2014). The therapeutic potential of ASC has been shown in several models of disease (Feisst et al., 2015). On the basis of these experimental studies, ASC entered clinical trials for diverse indications including musculoskeletal disorders, fistula, ulcers following peripheral artery disease, multiple sclerosis, myocardial infarction and stroke, COPD, and pulmonary fibrosis (can be reviewed at https://stemcellsportal.com/ifats-clinical-trials-view). Significant promise shown by ASC (Bateman et al., 2018) led to therapy progression to phase II and phase III clinical trials and regulatory approval for Crohn’s fistula (Galipeau and Sensebe, 2018; Olsen et al., 2018).

Since anti-inflammatory, anti-apoptotic, proliferative, angiogenic, immunomodulatory, and antioxidant effects displayed by stromal cells are attributed to secreted factors (Liang et al., 2014), cell-free stromal cell secretome can represent an alternative therapy for the treatment of pathologic conditions benefiting from stromal cell therapy. Secretome preparations can be standardized, generated and distributed in advance, and will not require specialized equipment/facilities currently needed for the isolation of ASC-containing stromal vascular fraction cells or storage of expanded stromal cells. In preclinical studies, we and others have demonstrated that ASC-conditioned media (ASC-CM) effectively suppresses a variety of pathologies ranging from bone loss (Li et al., 2018) and neurodegeneration (Fontanilla et al., 2015) to kidney (Bi et al., 2007) and lung injury (Lu et al., 2015). Methods of CM preparation for preclinical and early clinical studies were primarily tailored to the intended mode of administration while being influenced by somewhat fragmentary knowledge about the distribution of biological activity among various fractions. Unconcentrated CM, CM concentrates with low molecular weight (MW) cutoffs (Pawitan, 2014), and extracellular vesicle/exosome preparations (Giebel et al., 2017) were found to be effective in different preclinical and clinical studies. However, the future development of CM preparation as a therapeutic will rely on the ability to optimize the manufacturing process to balance maximizing bioactivity with the cost-efficient manufacturing, distribution, and storage of the final product. Whereas broad preclinical effects of CM preparations speak to their wide-ranging and robust therapeutic potential, the multifactorial nature of CM presents the challenge of product standardization, which necessitates elucidation of indication-relevant bioactive components in CM and the development of indication-relevant bioactivity tests.

We and others have recently shown that stromal cell CM effectively suppresses indices of lung injury *in vivo* (Ionescu et al., 2012; Lu et al., 2015). Lung injury has a complex pathology involving excessive inflammatory response, concomitant with the loss of endothelial and epithelial barrier function and subsequent lung cell death (Thompson et al., 2017). In the present study, we used endothelial responses relevant to lung injury, namely barrier dysfunction and apoptosis, to analyze partitioning of ASC-CM biological activities based on MW. Here, we show that the protection from apoptosis and barrier dysfunction is rendered by two non-overlapping fractions of ASC-CM. We found that barrier-protective properties of ASC-CM are preserved in CM subjected to concentration with low MW cutoff filters, while apoptosis-protective properties are significantly reduced by the concentration process.

## Materials and Methods

### Materials

Forty-kDa fluorescein isothiocyanate (FITC)-dextran, NaSH, and antibody to β-actin (1/10,000 dilution used) were purchased from Sigma (St. Louis, MO). Antibodies to cleaved caspase 3, phospho- and pan-p38, phospho- and pan-ERK, and phospho- and pan-c-Jun N-terminal kinase (pan-JNK) were from Cell Signaling (Beverly, MA) and were used at 1/1,000 dilution. CD81 and CD63 antibodies (1/1,000 dilution) and heme oxygenase 1 siRNA were from Santa Cruz Biotechnology (Dallas, TX). Heme oxygenase 1 antibody (1/1,000) and hepatocyte growth factor (HGF) siRNA were from Thermo Fisher Scientific (Waltham, MA). HRP-conjugated anti-rabbit and anti-mouse antibodies were from Cell Signaling and were used at 1/3,000. JNK inhibitor II and 3, 10, 50, and 100 kDa cutoff centrifugation filter inserts were from EMD Millipore (Billerica, MA). Carbon monoxide-releasing molecule 2 (CORM2) was from Tocris Bioscience (Minneapolis, MN).

### Cell Culture

All procedures for collecting human adipose tissue were approved by the Indiana University School of Medicine Institutional Review Board. Human and rat ASC were isolated from subcutaneous adipose tissue samples and characterized as described in Lu et al. (2015). Human pulmonary artery endothelial cells (HPAEC) were purchased from Lonza (Walkerville, MD) and used at passages 5–8. Rat lung microvascular endothelial cells (RLEC) were kindly provided by Dr. Irina Petrache (Schweitzer et al., 2011) (Indiana University) and used up to passage 16, at which transendothelial electrical resistance (TER) levels characteristic of endothelial monolayers were consistently observed. RLEC were maintained in DMEM-high glucose supplemented with 10% FBS and 1% penicillin–streptomycin. Both ASC and HPAEC were propagated using endothelial growth media 2-microvascular (EGM2-MV) (Lonza).

### ASC-CM Generation

Conditioned media from rat and human ASC were generated by incubating subconfluent ASC (250,000 cells/ml) with EGM-2MV for 48 h. To deplete ASC-CM of specific factors, 50 nM siRNA mixed with DharmaFECT 1 (Dharmacon, Lafayette, CO) was applied to 30% confluent ASC for 24 h.

### ASC-CM Manipulation

To heat-inactivate media, batches of ASC-CM and EGM-2MV were subjected to 30 min in boiling water bath, followed by 10 min centrifugation at 10,000 × *g* and collection of the supernatant. To deplete exosomes, batches of ASC-CM and EGM-2MV were subjected to 70 min ultracentrifugation at 100,000 × *g* using Sorvall ultracentrifuge, and the supernatant was collected and used for analyses. The same batches of heat-inactivated and exosome-depleted media were used to perform barrier dysfunction and apoptosis assays. For Western blot analysis, pellet from ultracentrifugation was dissolved in 1% sodium dodecyl sulfate (SDS) on PBS and analyzed with CD81 and CD63 antibodies. To fractionate media, ASC-CM and EGM-2MV were concentrated by centrifugation at 4,000 × *g* using 3, 10, 50, and 100 kDa filter inserts; 0.4 mg/ml solution of 40 kDa FITC-dextran was subjected to similar fractionation to verify the fractionation method. Fractionation was stopped when 40% of the initial volume remained.

### ELISA Analyses

Quantikine ELISA kits for human HGF and KGF were from R&D Systems (Minneapolis, MN). Vascular endothelial growth factor (VEGF) content was determined using sandwich ELISA with anti-human VEGF capturing and biotinylated detecting antibody and streptavidin-horse radish peroxidase complex (R&D Systems). ASC-CM was concentrated with 3 kDa filter (KGF) or diluted (HGF, VEGF) to allow detection in the linear range.

### Measurement of Transendothelial Permeability

TER was measured using Electrical Cell-Substrate Impedance Sensing (ECIS) (Applied Biophysics, Troy, NY) as described previously (Bogatcheva et al., 2009). HPAEC or RLEC plated on gold electrodes of ECIS array chambers were exposed to 1:1 mixture of EGM-2MV and test media for 48–72 h. In growth factor supplementation experiments, endothelial cells were exposed to the indicated concentration of growth factors for 48–72 h. At the end of the pre-incubation period, endothelial resistance reached 1,200–1,400 Ω for HPAEC and 1,800–2,000 Ω for RLEC, evident of monolayer confluence. Media were changed to basal media EBM-2 (Lonza) 2 h prior to the beginning of TER recording.

### Western Immunoblotting

HPAEC or RLEC grown in 12-well plates were exposed to 1:1 mixture of EGM-2MV and test media for 72 h; media was changed to EBM-2 1 h prior to the beginning of stimulation. Cells were stimulated with 2 ng/ml tumor necrosis factor (TNF) for the times indicated in the figure legends. ASC grown in 12-well plates were treated with siRNA as described in the figure legends. Protein extracts were prepared by lysing cells with 1% SDS-containing buffer and separated on 4–20% polyacrylamide gels followed by transfer to nitrocellulose membrane. After staining with specific antibodies, a signal was developed, imaged, and quantified with Bio-Rad imaging system.

### Statistical Analysis

Repeated measures one-way ANOVA (GraphPad Prism 6) or one-way ANOVA with Tukey *post hoc* was used to analyze TER recordings. One-way ANOVA with Tukey *post hoc* or *t*-test with Welch’s correction (unequal variance) was used to analyze Western blot and ELISA results. A probability value of < 0.05 was considered statistically significant.

## Results

### Endothelial-Protective Factors in ASC-CM Are Detected in > 50 kDa Fraction, Whereas Apoptosis-Protective Factors Are Detected in < 3 kDa Fraction

**FIGURE 1:**
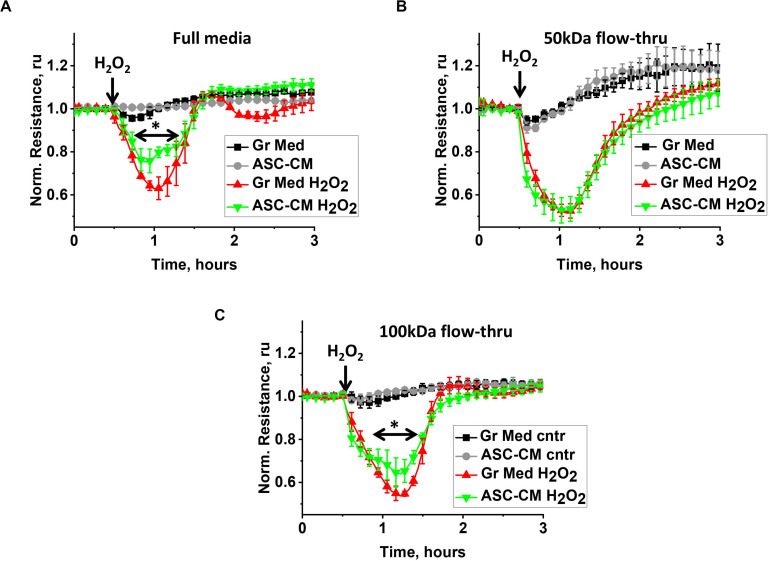
ASC-CM factors preserving transendothelial permeability partition with MW > 50 kDa. HPAEC grown on gold electrodes of ECIS arrays were exposed to **(A)** the original growth media (black, red) or ASC-CM (gray, green), **(B)** 50 kDa flow-through fractions of the growth media (black, red) or of ASC-CM (gray, green), and **(C)** 100 kDa flow-through fractions of the growth media (black, red) or of ASC-CM (gray, green). Media were removed after 72 h of pretreatment, and HPAEC were challenged with 250 μM H_2_O_2_ (red, green) or vehicle (black, gray). Data are presented as mean ± SEM from three parallel recordings; the results were reproduced in at least three independent experiments. ^∗^Repeated measurement one-way ANOVA detected significant differences in **(A)** responses of HPAEC exposed to unmanipulated ASC-CM (green) when compared to unmanipulated growth media (red) and **(C)** responses of HPAEC exposed to 100 kDa flow-through of ASC-CM (green) when compared to 100 kDa flow-through of growth media (red).

We had previously shown that preconditioning of the endothelium with ASC-secreted factors protects it from hyperpermeability and activation of pro-apoptotic pathways; a protective effect was not detected when the endothelium was preconditioned with dermal fibroblast-secreted factors (Lu et al., 2015). To understand how the biological activity of ASC-CM is partitioned among different MW fractions, we compared the abilities of the original CM and its various flow-through fractions to attenuate H_2_O_2_-induced endothelial barrier dysfunction and TNFα-induced endothelial apoptosis. Figure 1A shows the typical response of the control endothelium to H_2_O_2_ stimulation. H_2_O_2_ challenge of monolayers pretreated with control media led to a dramatic decrease of TER within the first 15–30 min, followed by the period of barrier restoration. HPAEC pretreated with unmanipulated ASC-CM also manifested a decrease in TER, however, restoration of barrier function occurred faster (Figure 1A), minimizing the duration and severity of endothelial barrier leakage. Exposure of the endothelium to the ASC-CM flow-through fractions obtained with MW cutoffs of 50 kDa (Figure 1B) or less (not shown) did not result in endothelial protection from the barrier-disruptive effect of H_2_O_2_. Only the 100-kDa flow-through fraction of ASC-CM manifested a barrier-protective activity (Figure 1C).

**FIGURE 2:**
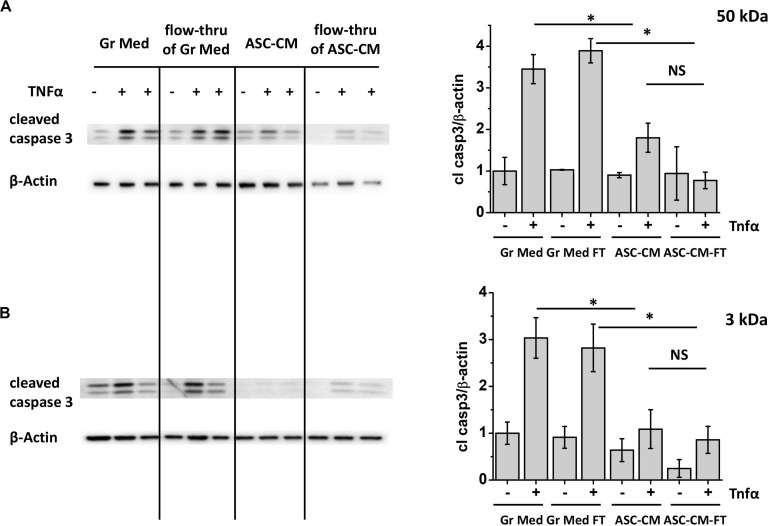
ASC-CM factors preventing endothelial apoptosis partition with MW < 3 kDa. HPAEC pretreated with the original growth media and ASC-CM, or the 50- **(A)** or 3-kDa **(B)** flow-through fractions of growth media and ASC-CM were challenged with 2 ng/ml TNFα for 4 h. Cell lysates were analyzed with antibodies to cleaved caspase 3 and β-actin (loading control). Data from three independent experiments were pooled and presented as cleaved caspase 3/β-actin ratio mean ± SEM. One-way ANOVA with Tukey *post hoc* was used to detect whether differences between indicated columns are significant ^∗^(*p* < 0.05) or not significant (NS).

Figure 2A shows the typical response of control media-pretreated HPAEC to TNFα stimulation. Challenge of control monolayers with TNFα led to a marked increase in caspase 3 cleavage, indicative of pro-apoptotic activation of endothelium. This process was attenuated in monolayers pretreated with unmanipulated ASC-CM. Exposure of the endothelium to the ASC-CM flow-through fractions obtained with MW cutoffs of 50 kDa resulted in significant endothelial protection from the pro-apoptotic induction by TNFα (Figure 2A). Moreover, 10 (not shown) and 3 kDa flow-through fractions were similarly effective in the prevention of pro-apoptotic response to TNFα (Figure 2B).

### Heat Inactivation and Depletion of Extracellular Vesicles From ASC-CM Affects Endothelial Barrier Protection but Not Apoptosis Protection

**FIGURE 3:**
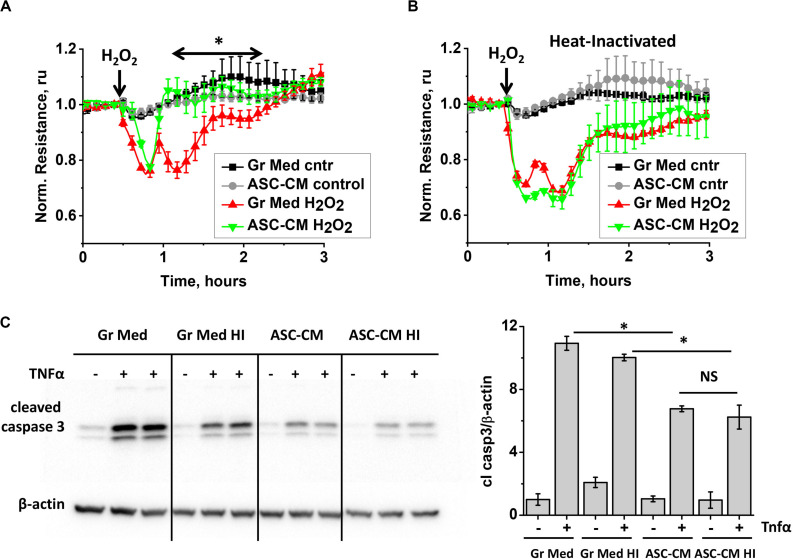
ASC-CM factors preserving transendothelial permeability are heat-sensitive, whereas factors preventing endothelial apoptosis are not. **(A,B)** HPAEC grown on gold electrodes of ECIS arrays were exposed to **(A)** the original growth media (black, red) or ASC-CM (gray, green) and **(B)** heat-inactivated growth media (black, red) or ASC-CM (gray, green). After 72 h of preconditioning, media were substituted with EBM-2, and HPAEC were challenged with 250 μM H_2_O_2_ (red, green) or vehicle (black, gray). Shown are the means ± SEM of three parallel recordings; the results were reproduced in at least three independent experiments. **(C)** HPAEC pretreated with the original or heat-inactivated (HI) growth media and ASC-CM were challenged with 2 ng/ml TNFα for 4 h. Cell lysates were analyzed with antibodies to cleaved caspase 3 and β-actin (loading control). Data from three independent experiments were pooled and presented as cleaved caspase 3/β-actin ratio mean ± SEM. One-way ANOVA with Tukey *post hoc* was used to detect whether differences between indicated columns are significant ^∗^(*p* < 0.05) or not significant (NS).

To gain more information about the nature of bioactive components in ASC-CM, we subjected ASC media to several manipulations. First, we heat-inactivated ASC–CM and assessed its ability to confer endothelial protection from barrier dysfunction and apoptosis. Comparison of responses of HPAEC monolayers pretreated with unmanipulated (Figure 3A) and heat-inactivated (Figure 3B) ASC-CM shows that heat inactivation results in a loss of ASC-CM’s ability to protect the endothelium from barrier dysfunction.

Remarkably, heat inactivation of ASC-CM did not void its ability to protect the endothelium from apoptosis (Figure 3C), suggestive of the heat-stable nature of apoptosis-protective factors.

**FIGURE 4:**
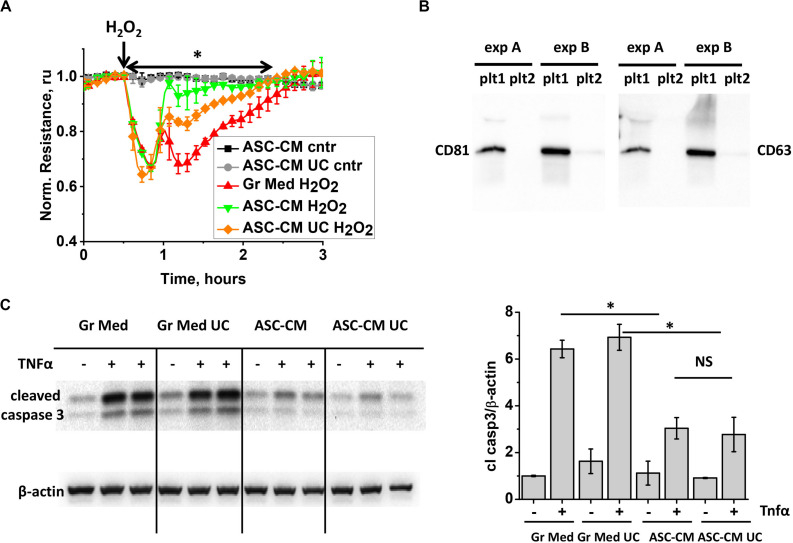
ASC-CM factors preserving transendothelial permeability are partially associated with extracellular vehicles, whereas factors preventing endothelial apoptosis are not. **(A)** HPAEC grown on gold electrodes of ECIS arrays were exposed to the original growth media (black, red), ASC-CM (green), or EV-depleted supernatant of ASC-CM subjected to 100,000 × *g* ultracentrifugation (gray, orange). After 72 h of preconditioning, media were substituted with EBM-2, and HPAEC were challenged with 250 μM H_2_O_2_ (red, green, orange) or vehicle (black, gray). Shown are the means ± SEM of three parallel recordings; the results were reproduced in at least three independent experiments. ^∗^Repeated measurement one-way ANOVA detected significant differences between responses of HPAEC exposed to the original ASC-CM (green) and ASC-CM subjected to ultracentrifugation (orange). **(B)** ASC-CM were subjected to 10,000 × *g* centrifugation to remove the cell debris, followed by 100,000 × *g* ultracentrifugation, and a subsequent round of 100,000 × *g* ultracentrifugation to remove EV. Pellets from the first (plt1) and second (plt2) rounds of 100,000 × *g* ultracentrifugation were analyzed by Western blotting with CD81 and CD63 antibodies. Results from two independent experiments **(A,B)** are presented. **(C)** HPAEC pretreated with the original and ultracentrifuged (UC) growth media and ASC-CM were challenged with 2 ng/ml TNFα for 4 h. Cell lysates were analyzed with antibodies to cleaved caspase 3 and β-actin (loading control). Data from three independent experiments were pooled and presented as cleaved caspase 3/β-actin ratio mean ± SEM. One-way ANOVA with Tukey *post hoc* was used to detect whether differences between indicated columns are significant ^∗^(*p* < 0.05) or not significant (NS).

To further characterize the active components in ASC-CM, we subjected ASC-CM to ultracentrifugation at 100,000 × *g* to deplete extracellular vesicles (EV), which have been shown to mediate many beneficial activities of stromal cells (Fujita et al., 2018). Exposure of the endothelium to the control growth media subjected to ultracentrifugation did not affect its response to H_2_O_2_ (data not shown). However, exposure of the endothelium to EV-depleted ASC-CM revealed that its barrier-protective potency is significantly, but not completely, attenuated as a result of ultracentrifugation (Figure 4A). To ensure that the duration of ultracentrifugation was sufficient to remove EV from ASC-CM, we subjected the supernatant from the first round of ultracentrifugation to a second, consecutive round. The pellets from both rounds were collected and analyzed for the presence of exosomal markers CD81 and CD63 (tetraspanin). CD81 and CD63 staining was positive in the pellet resultant from the first, but not the second ultracentrifugation (Figure 4B), suggesting near complete depletion of EV from ASC-CM with one round of ultracentrifugation.

When pro-apoptotic responses of HPAEC were assessed in monolayers pretreated with ASC-CM subjected to ultracentrifugation, we found that EV depletion did not decrease ASC-CM’s ability to prevent activation of caspase 3 cleavage (Figure 4C).

### Endothelial-Protective Factors in ASC-CM Are Species-Specific, While Apoptosis-Protective Factors Are Not

**FIGURE 5:**
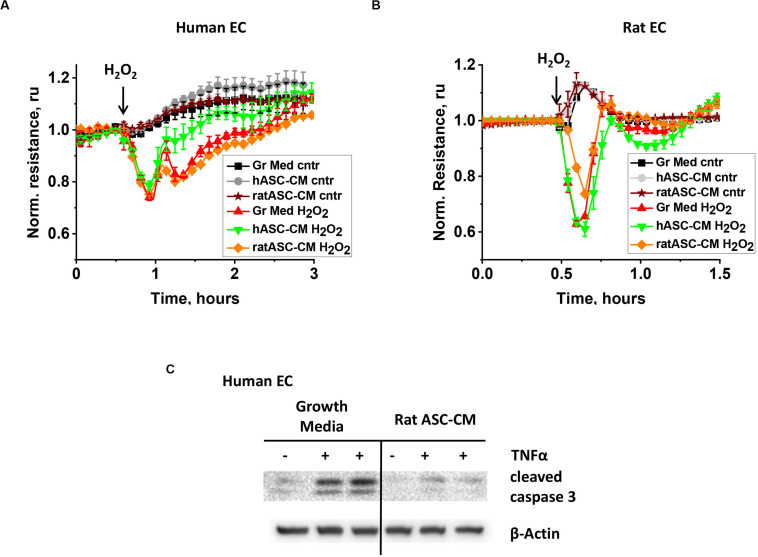
ASC-CM factors protecting from barrier dysfunction are species-specific, whereas anti-apoptotic factors are not. HPAEC **(A)** or RLEC **(B)** grown on gold electrodes of ECIS arrays were exposed to the original growth media (black, red), human ASC-CM (gray, green), or rat ASC-CM (brown, orange). Media were removed after 72 h of endothelial cell exposure, and HPAEC/RLEC were challenged with 250 μM H_2_O_2_ (red, green, orange) or vehicle (black, gray, brown). Shown are the means ± SEM of three parallel recordings. **(C)** HPAEC were pretreated with growth media or rat ASC-CM. After 72 h, media was substituted with EBM-2; HPAEC were challenged with 2 ng/ml TNFα for 4 h. Cell lysates were analyzed with antibodies to cleaved caspase 3 and β-actin (loading control).

To gain further information about the nature of bioactive factors in ASC-CM, we assessed whether barrier protection and apoptosis protection are exerted in a species-specific manner. First, human endothelial cells were pretreated with CM from rat and human ASC and analyzed for H_2_O_2_-induced hyperpermeability. Unlike CM from same-species ASC, rat ASC-CM did not confer barrier protection to human endothelium (Figure 5A). Next, rat endothelial cells were pretreated with human and rat ASC-CM. While rat ASC-CM exposure attenuated RLEC response to H_2_O_2_, human ASC-CM did not (Figure 5B).

When a similar experiment was conducted to assess the apoptosis-protective properties of xenogeneic ASC-CM, rat ASC-CM conferred protection from TNFα-induced apoptosis in human endothelium (Figure 5C).

### Barrier-Protective Properties of ASC-CM Can Be Partially Attributed to the Secreted KGF and HGF

**FIGURE 6:**
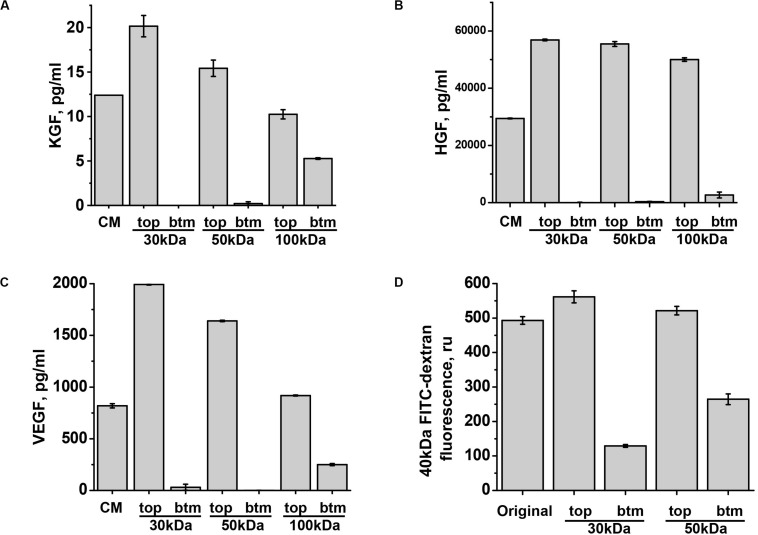
KGF, HGF, and VEGF partition in the fraction with MW exceeding 50 kDa. **(A–C)** ASC-CM was generated over the period of 48 h. KGF, HGF, and VEGF concentrations were determined in the original ASC-CM (CM), and top (tp) and bottom (bt) fractions generated by filtration through indicated MW cutoff membranes. Data from three independent experiments are presented. **(D)** 40 kDa FITC-dextran was filtered *via* indicated MW cutoff membranes; fluorescence levels of the original solution and top and bottom fractions are presented.

We next focused our attention on three factors which are known at certain concentrations to improve endothelial barriers: VEGF (Mirzapoiazova et al., 2006), KGF (Gillis et al., 1999), and HGF (Liu F. et al., 2002). To understand in which fraction of ASC-CM these factors would partition, we assessed the levels of KGF, HGF, and VEGF A in 30, 50, and 100 kDa flow-through fractions. Surprisingly, detectable amounts of KGF (MW ∼19 kDa) and VEGF 121 and VEGF 165 (estimated MW ∼20 kDa, but may migrate as 30 kDa due to glycosylation) appeared in 100 kDa flow-through only (Figures 6A,C). HGF (estimated MW for α- and β-chains are 54 and 26 kDa, respectively) also partitioned with higher MW than expected, with only minor fraction detected in 100 kDa flow-through (Figure 6B). To ascertain that fractionation *via* 30 and 50 kDa cutoff filters occurred according to specified MW, we subjected 40 kDa FITC-dextran to the filtration. As expected from the product with the normal distribution of MW peaking at 40 kDa, passage of some fluorescent material was detected in 30 kDa flow-through; fluorescence level was higher in 50 kDa flow-through (Figure 6D). Altogether, these data show that KGF, VEGF, and HGF fractionate as bigger MW complexes. Importantly, analysis of flow-through rendering barrier protection (Figures 1A–C) indicated that 50 kDa and less flow-through fractions deficient in KGF, VEGF, and HGF do not possess barrier-protective properties.

**FIGURE 7:**
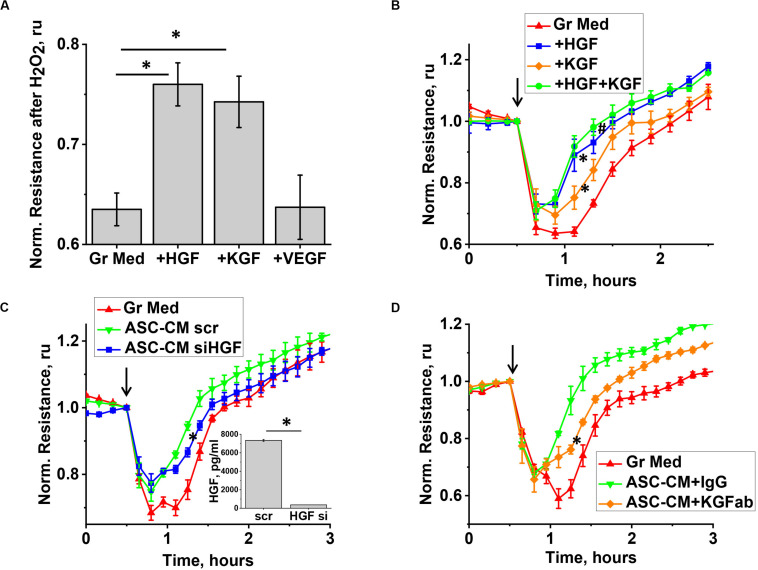
KGF and HGF protect the endothelium from H_2_O_2_-induced barrier dysfunction. **(A,B)** HPAEC grown on gold electrodes of ECIS arrays were exposed to the original growth media (B-red), or growth media supplemented with KGF (B-orange), HGF (B-blue), KGF + HGF (B-green), or VEGF concentrations detected in ASC-CM conditioned for 48 h (see Figure 6). Media supplemented with factors were removed after 48 h of pretreatment, and HPAEC were challenged with 250 μM H_2_O_2_. For **(A)**, TER values were pooled from three independent experiments, with at least two parallel recordings each, and calculated as fold decrease in TER 30 min after H_2_O_2_ addition. **(C)** HPAEC grown on gold electrodes of ECIS arrays were exposed to the original growth media, or ASC-CM generated by ASC treated with scrambled siRNA, or HGF siRNA. Media were removed after 48 h of pretreatment, and HPAEC were challenged with 250 μM H_2_O_2_. Inset shows the concentration of HGF in ASC-CM collected from scrambled RNA-treated and HGF siRNA-treated ASC. **(D)** HPAEC grown on gold electrodes of ECIS arrays were exposed to the original growth media, or ASC-CM pre-incubated with control IgG or KGF neutralizing antibody. Media were removed after 48 h of pretreatment, and HPAEC were challenged with 250 μM H_2_O_2_. **(A)** ^∗^Differences detected with one-way ANOVA with Tukey *post hoc* (*p* < 0.05). **(B)** Repeated measurements ANOVA detected differences between responses of HPAEC exposed to growth media supplemented with either KGF or HGF (^∗^), and the original growth media. Differences were also detected between responses of HPAEC exposed to media supplemented with KGF and KGF + HGF (#). **(C,D)** ^∗^ Repeated measurements ANOVA detected differences between responses of HPAEC exposed to unmanipulated ASC-CM or ablated ASC-CM. (**C**, inset) ^∗^ Differences were detected by *t*-test (*p* < 0.05).

We next proceeded to test whether levels of KGF, VEGF, and HGF detected in ASC-CM would be sufficient to confer barrier protection exerted by ASC-CM. As data of literature suggest that some of the factors, such as KGF, can be associated with EV while secreted by stromal cells (Zhu et al., 2014), we first assessed whether ELISA assays employed to assess factor levels in ASC detect soluble or total levels of factors. For that, we compared the levels of VEGF, KGF, and HGF in the original ASC-CM and ASC-CM depleted by ultracentrifugation. We did not observe decreases in VEGF, HGF, and KGF levels after ultracentrifugation (data not shown), suggesting that the levels indicated in Figure 6 reflect levels of soluble factors only and not levels of factors associated with EVs. We next supplemented EGM-2MV with the concentrations of soluble VEGF, HGF, and KGF found in ASC-CM (1 ng/ml, 30 ng/ml, and 10 pg/ml, respectively) and pretreated the endothelium for 72 h. Our data indicated that preconditioning of HPAEC with VEGF did not protect the endothelium from H_2_O_2_-induced barrier disruption (Figure 7A). However, exposure of endothelium to KGF and HGF significantly suppressed H_2_O_2_-induced decrease in TER (Figure 7B). In concert with these data, ASC with HGF siRNA knockdown yielded ASC-CM with attenuated barrier-protective properties (Figure 7C). Similar to HGF depletion, KGF neutralization with anti-KGF antibody attenuated the barrier-protective properties of ASC-CM (Figure 7D).

### Apoptosis-Protective Properties of ASC-CM Can Be Partially Attributed to the Generated Carbon Monoxide

**FIGURE 8:**
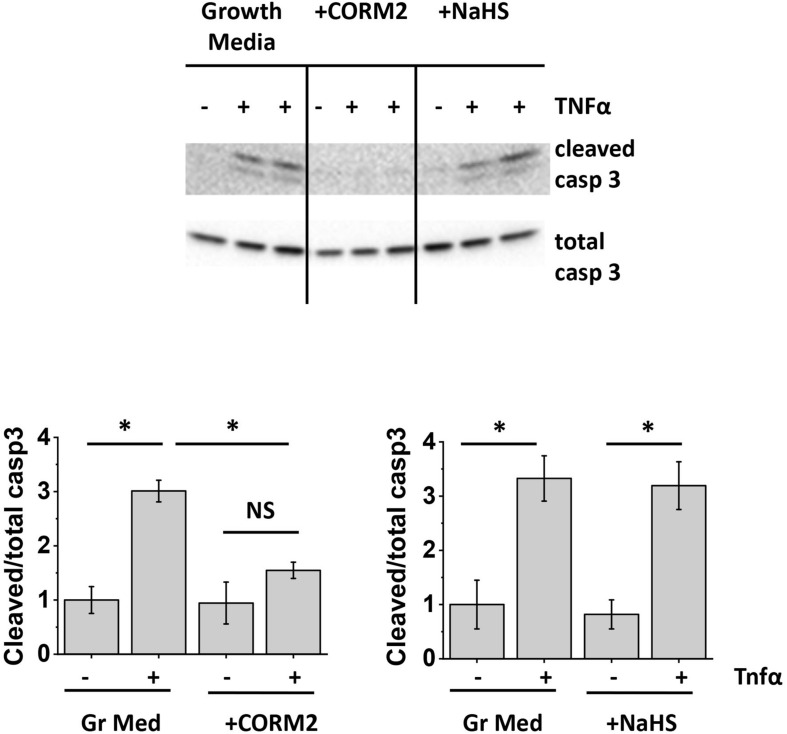
Carbon monoxide protects the endothelium from apoptosis. HPAEC were exposed to the original growth media or growth media supplemented with 50 μM CORM2 (CO donor) or 50 μM NaHS (H_2_S donor). After 48 h, media was removed; cells were stimulated with 2 ng/ml TNFα. Cell lysates were analyzed with antibodies to cleaved and total caspase 3. Data from three independent experiments were pooled and presented as cleaved/total caspase 3 ratio mean ± SEM. One-way ANOVA with Tukey *post hoc* was used to detect whether differences between indicated columns are significant ^∗^(*p* < 0.05) or not significant (NS).

Since our data indicated that apoptosis protection by ASC-CM is conveyed by heat-stable, non-species-specific factors of low MW, we next assessed the ability of two stable gases with known anti-apoptotic properties, namely carbon monoxide (Liu X.M. et al., 2002; Almeida et al., 2012; Li et al., 2012) and hydrogen sulfide (Sivarajah et al., 2009; Wu et al., 2015), to protect HPAEC from TNFα-induced apoptosis. CO- and H_2_S-generating enzymes are known to be expressed in mesenchymal stromal cells (Zarjou et al., 2011; Gambari et al., 2017). To assess whether CO and H_2_S can mimic the beneficial effect of ASC-CM, we preconditioned HPAEC with CO donor CORM2 (Babu et al., 2017) and H_2_S donor NaHS (Wu et al., 2015). Figure 8A shows that CORM2 exposure, but not NaHS exposure, significantly attenuated endothelial caspase 3 cleavage in response to TNFα.

**FIGURE 9:**
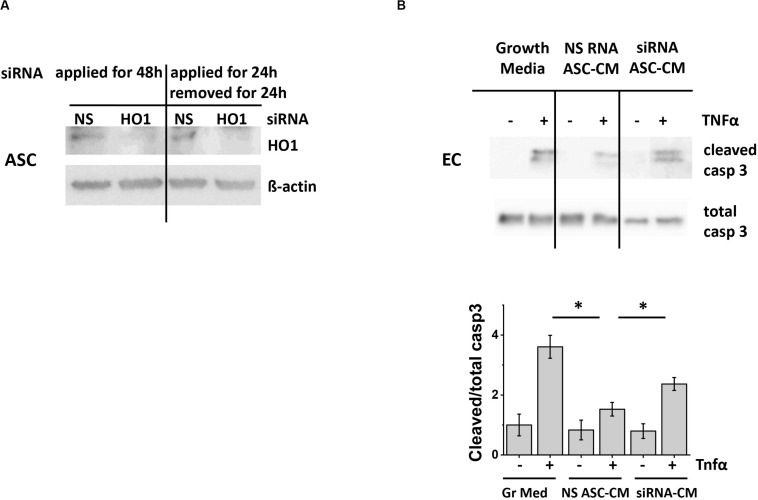
ASC with suppressed heme oxygenase 1 expression generate ASC-CM with reduced apoptosis-protective potency. **(A)** Left: ASC were treated continuously with 50 nM of non-specific (NS) or heme oxygenase 1 (HO-1)-specific siRNA for 48 h. Right: Treatment was applied for 24 h, followed by a 24 h incubation in siRNA-free media. Cell lysates were analyzed with antibodies to heme oxygenase 1 and β-actin (loading control). **(B)** ASC-CM was generated from ASC treated for 24 h with 50 nM non-specific RNA (NS RNA) or heme oxygenase 1-specific siRNA (siRNA), followed by siRNA removal and 24 h conditioning with media. HPAEC pre-incubated for 48 h with growth media or ASC-CM were stimulated with 2 ng/ml TNF. Cell lysates were analyzed with antibodies to cleaved and total caspase 3. Data from three independent experiments were pooled and presented as cleaved/total caspase 3 ratio mean ± SEM. ^∗^Significance between TNF-stimulated groups assessed by *t*-test with Welch’s correction as indicated (*p* < 0.05).

To generate ASC-CM with decreased amount of carbon monoxide, we knocked down CO-generating enzyme heme oxygenase 1 (HO-1) with specific siRNA. We first assessed whether the suppression of HO-1 will be stable after the removal of siRNA for the period required to generate ASC-CM. Figure 9A shows that ASC demonstrated sustained changes in HO-1 expression 24 h after the removal of siRNA. These conditions were applied to generate ASC-CM free of HO-1 siRNA, which could have had direct effects on endothelial monolayers.

To assess the effect of CO depletion on ASC-CM apoptosis-protective properties, we preconditioned HPAEC with CM from ASC subjected to HO-1 knockdown. Control ASC-CM pretreatment led to the significant suppression of caspase 3 activation in HPAEC (Figure 9B); CM from ASC treated with HO-1 siRNA displayed attenuated potency. Altogether, these data suggested that carbon monoxide represents one of the factors conferring ASC-CM-mediated endothelial protection from apoptosis.

### Protection of Endothelium From Apoptosis Is Dependent on c-Jun N-Terminal Kinase Inactivation

**FIGURE 10:**
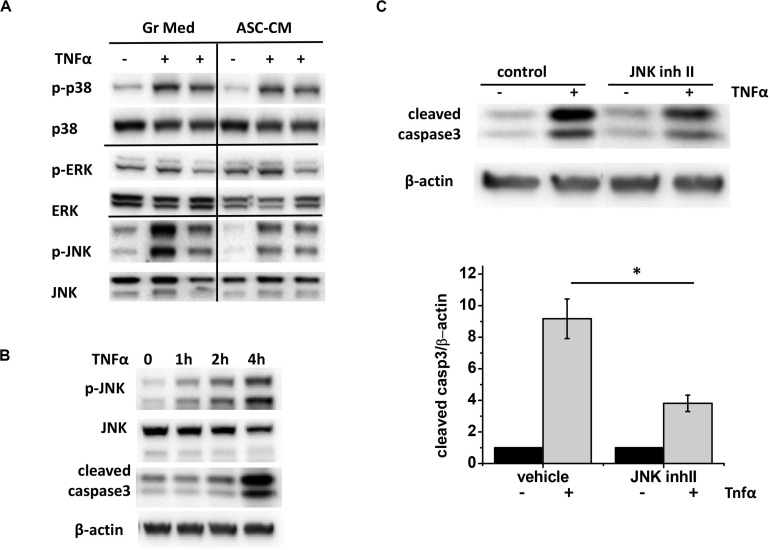
ASC-CM exposure suppresses JNK activation, which precedes and contributes to caspase activation in response to TNFα in the endothelium. **(A)** HPAEC exposed to growth media or ASC-CM were challenged with 2 ng/ml TNFα for 4 h. Cell lysates were analyzed with antibodies to phospho- and pan-p38, phospho- and pan-ERK, and phospho- and pan-JNK. **(B)** HPAEC were stimulated with 2 ng/ml TNFα for the times indicated. Cell lysates were analyzed with antibodies to p-JNK, JNK, cleaved caspase 3, and β-actin (loading control). **(C)** HPAEC pretreated with vehicle control or 5 μM JNK inhibitor II for 30 min were treated with 2 ng/ml TNFα for 4 h. Data from three independent experiments were pooled and presented as cleaved caspase 3/β-actin ratio, normalized to no TNF control. ^∗^*t*-test with Welch’s correction was used to detect significant differences (*p* < 0.05).

To learn about which pro-apoptotic pathways are targeted by apoptosis-protective factors in ASC-CM, we first analyzed endothelial MAP kinases activated in response to TNFα. We observed that TNFα stimulation leads to robust phosphorylation of p38 and JNK (Figure 10A) and very limited phosphorylation of ERK. However, when the effect of ASC-CM exposure was analyzed, only JNK phosphorylation was dramatically suppressed. Activation of JNK preceded activation of caspase 3 cleavage (Figure 10B), suggesting that JNK activation lays upstream of the activation of pro-apoptotic caspase cascades. To check if inhibition of JNK would protect from TNFα-induced apoptosis, we pretreated HPAEC with JNK inhibitor II. Indeed, we observed that in the presence of JNK inhibitor, cleavage of caspase 3 in response to TNF was significantly attenuated (Figure 10C).

**FIGURE 11:**
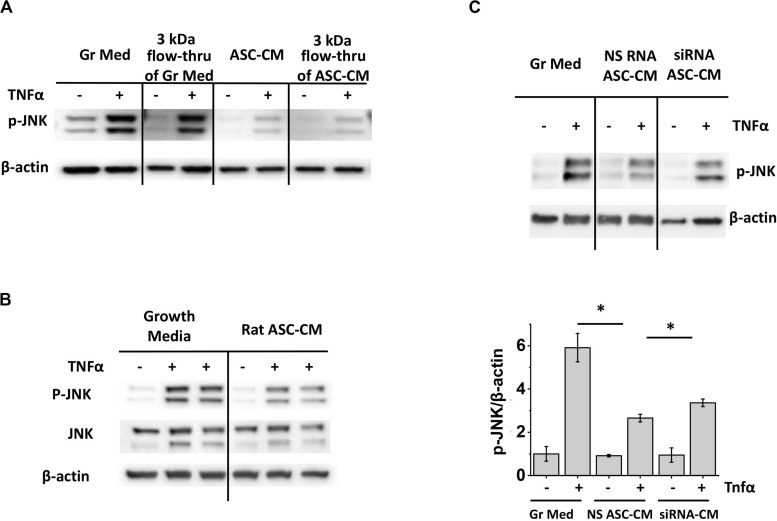
Inhibition of JNK activation is mediated by small MW factors in ASC-CM which are not species-specific. Depletion of heme oxygenase 1 in ASC attenuates ASC-CM ability to inhibit JNK activation. **(A)** HPAEC pretreated with the original growth media and ASC-CM or the 3 kDa flow-through fractions of growth media and ASC-CM were challenged with 2 ng/ml TNFα for 4 h. **(B)** HPAEC pretreated with growth media or rat ASC-CM were challenged with 2 ng/ml TNFα for 4 h. **(C)** HPAEC pretreated with growth media or ASC-CM generated by non-specific RNA-treated ASC (NS RNA ASC-CM) or heme oxygenase 1 siRNA-treated ASC (siRNA ASC-CM) were challenged with 2 ng/ml TNFα for 4 h. Cell lysates were analyzed with antibodies to p-JNK and β-actin (loading control). Data from three independent experiments were pooled and presented as cleaved caspase 3/β-actin ratio mean ± SEM. ^∗^Significance between TNF-stimulated groups assessed by *t*-test with Welch’s correction as indicated (*p* < 0.05).

To ascertain that the ASC-CM factors suppressing JNK activation are of the same nature as the factors suppressing caspase 3 activation, we first preconditioned HPAEC with a 3-kDa flow-through of ASC-CM. Figure 11A shows that 3 kDa flow-through effectively suppressed JNK phosphorylation, similar to suppression of caspase 3 cleavage shown previously (Figure 2B). We next verified whether xenogeneic ASC-CM would have an effect similar to allogeneic ASC-CM. Preconditioning of HPAEC with rat ASC-CM resulted in effective suppression of JNK phosphorylation (Figure 11B), suggesting that inhibition of the JNK pathway and inhibition of the pro-apoptotic pathway are rendered by a similar class of factors in ASC-CM. Finally, we preconditioned HPAEC with the CM from HO-1-depleted ASC and assessed JNK phosphorylation in response to TNFα. As was earlier shown with caspase 3 activation, CM from HO-1-depleted ASC displayed attenuated ability to suppress JNK activation (Figure 11C).

## Discussion

The progress of stromal cells to clinical studies testing application in multiple diseases highlighted several issues that may limit the widespread adoption for clinical use. These include issues with consistency of cell preparations for autologous treatments and logistical issues associated with cell storage, distribution, and bioactivity of cryopreserved cell preparations, whether autologous or allogeneic. Although closed system isolation devices, allowing isolation and readministration of autologous ASC-containing products at the point of care, are available for clinical use (Nordberg and Loboa, 2015), issues of patient-to-patient variability and safety concerns pertinent to the risk of thromboembolism remain (Toyserkani et al., 2017). The search for cell-free alternatives with potential advantages in safety, material handling, and bioactivity control was undertaken based on the discovery that stromal cell secretome manifested a significant portion of the effects attributed to stromal cells. The therapeutic potential of factors secreted by stromal cells was noted in multiple preclinical models of diseases, including acute conditions (Parekkadan et al., 2007; Cho et al., 2012; Ionescu et al., 2012; Lu et al., 2015; Pouya et al., 2018) for which the application of the off-the-shelf biologic can be of direct benefit as the product could be administered without delay and would exert immediate bioactivity, which may be critical in acute care settings such as stroke or myocardial infarction. To develop CM formulation translatable to clinical use, understanding of the effects of the manufacturing process on the product cost, logistics of storage and handling, and bioactivity is critically important. That is why, even though preclinical (Bi et al., 2007; Yamagata et al., 2013; Suto et al., 2016) and some clinical (Zhou et al., 2013; Dahbour et al., 2017) studies show the ability of unconcentrated CM to exert beneficial effects and limit pathological conditions, significant effort is devoted to analyze the effects of CM lyophilizates (Fukuoka et al., 2017; Katagiri et al., 2017) and EV concentrates (Kordelas et al., 2014; Nassar et al., 2016). Formulation of the particular therapeutic is likely to be optimized for specific clinical use; therefore, elucidation of the nature of bioactive factors is of utmost importance for the preservation of therapeutic potential during the manufacturing process and will be of utmost significance to gaining regulatory approval. Here, we used an unbiased approach to analyze biological activities in different fractions of the original CM, to gain information about the nature of bioactive factors and, most importantly, aid in understanding how routine procedures used in CM manufacturing, such as concentration utilizing different MW filters, can affect the biological activity of the CM product.

This study is not the first attempt to identify the fraction of stromal cell CM with specific biologic activity. As one can expect, the MW of active fraction depends on the nature of activity for which it is tested for. For example, the ability to preserve pancreatic islets was assigned to 10–30 and > 50 kDa fractions of ASC-CM (Kasahara et al., 2013). The ability to promote macrophage shift to anti-inflammatory phenotype was assigned to < 3 and 50–100 kDa fractions of CM (Ylostalo et al., 2012). On the contrary, the ability to reduce myocardial infraction was seen only in the CM fraction of > 1,000 kDa (Timmers et al., 2007). To aid in understanding endothelial-specific effects with the potential to apply this knowledge for treatment of vascular leak and endothelial damage associated with acute lung injury, we used endothelial barrier dysfunction and apoptosis as two easily quantifiable readouts. Employing human pulmonary endothelial cells as a reporting system, we assessed (1) barrier dysfunction in response to H_2_O_2_, mimicking endothelial response to oxidative burst, and (2) activation of pro-apoptotic pathways in response to TNFα, mimicking endothelial apoptotic response to cytokine storm. Although an early work (Petrache et al., 2003) failed to detect significant HPAEC apoptosis in response to TNFα, later studies showed consistent activation of pro-apoptotic caspase 3 (Lu et al., 2015) and dramatic increase in TUNEL-positive HPAEC in response to TNFα (Koh et al., 2007; Bae and Rezaie, 2008). Using readouts of H_2_O_2_-induced barrier dysfunction and TNFα-induced caspase 3 cleavage in HPAEC treated with different fractions of ASC-CM, we showed that barrier-protective and apoptosis-protective activities of ASC-CM are carried by its two distinct fractions, > 50 and < 3 kDa, respectively.

Analysis of the potential involvement of HGF and KGF, earlier shown to be secreted by stromal cells (Cai et al., 2007; Lafosse et al., 2016) and known to affect barrier function (Gillis et al., 1999; Liu F. et al., 2002), revealed that (1) these factors fractionate with MW exceeding 100 kDa and (2) levels of factors present in ASC-CM are sufficient to confer barrier resistance to H_2_O_2_ upon preconditioning. Ablation of either factor with neutralizing antibody or siRNA knockdown rendered CM with attenuated ability to protect the endothelium. Although the levels of HGF and KGF determined in CM by ELISA were not affected by CM centrifugation and therefore represented the levels of soluble factors, we cannot exclude the existence of exosome-associated factors with similar biologic activity. Consistently, the barrier-protective activity of CM can be partially attributed to extracellular vesicle/exosome fraction of CM. This fraction is also known to contain vast cargo of bioactive proteins and microRNA, whose particular analysis lies beyond the scope of this study.

Examination of the apoptosis-protecting activity of CM revealed heat-insensitive, non-species-specific, and small MW nature of these factors. Our attention was drawn to the possible role for the secreted gases; two of them, hydrogen sulfide and carbon monoxide, are known to be stable and anti-apoptotic (Almeida et al., 2012; Wu et al., 2015). Preconditioning of the endothelium with donors of H_2_S and CO revealed that only CORM2, the CO donor, is able to protect the endothelium from TNFα-induced apoptosis. In the absence of direct methods to measure CO production in CM, our next step was to ablate the CO-generating enzyme, heme oxygenase 1, in ASC. Our data showed that knockdown of heme oxygenase 1 decreased the apoptosis-protective potency of ASC-CM, suggesting that carbon monoxide generation by ASC is important for the anti-apoptotic activity exhibited by ASC or ASC unmanipulated secretome.

Further examination into the apoptosis-mediating mechanisms affected by CM preconditioning revealed the causative nature of JNK phosphorylation in the endothelium. The inhibitor used in the study is known to suppress both JNK1 and JNK2 activity. A recent study showed that only JNK1 possesses pro-apoptotic activity in the lung (Tan et al., 2020). Importantly, TNFα-induced JNK phosphorylation was suppressed by ASC-CM in the same heat-insensitive, non-species-specific, and low MW factor-dependent manner as TNFα-induced caspase 3 activation, prompting the examination of the role of carbon monoxide. Finally, we had shown that knockdown of heme oxygenase 1 in ASC reduced ASC-CM’s ability to inhibit JNK phosphorylation. Altogether, these data helped pinpoint carbon monoxide as one of the factors contributing to ASC-CM’s anti-apoptotic activity and delineate the pro-apoptotic pathways attenuated by ASC-CM in the endothelium. One of the limitations of the study is that the nuclear factor kappa B (NF-κB) pathway, contributing to TNFα response (Kempe et al., 2005), was not studied, whereas this pathway is known to protect cells from apoptotic death, in particular, by downregulating JNK signaling (De Smaele et al., 2001). Previously, proteomic analysis of exosomes released from serum-starved and oxygen-deprived MSC showed enrichment in NF-κB-regulating nodes; in the same study, MSC’s exosome ability to induce endothelial angiogenesis was shown to be NF-κB-dependent (Anderson et al., 2016). As our study shows that protection from apoptosis is mediated by the exosome-free fraction of ASC-CM, it would be interesting to see how TNFα-induced endothelial NF-κB signaling is affected by ASC-CM manipulation.

Summarizing the impact of our data for the development of application-specific CM preparations, we have to emphasize that although the barrier-protective activity of CM will be preserved in preparations generated by the concentration with low MW cutoff filters or ultracentrifugation, a significant proportion of apoptosis-protective activity will be lost. Nonetheless, the concentration of CM is the most likely scenario to be followed by large-scale manufacturers for clinical purposes; therefore, emphasis of future studies should be on the barrier-protective and immunomodulatory activities of fractions preserved after the concentration of CM with low MW cutoff filters. In the context of our data, standardization criteria for clinical applications aiding to preserve endothelial barrier should include assessment of levels of HGF and KGF in CM preparations. Our data regarding low MW components of secretome with anti-apoptotic activity, although of limited relevance to the development of CM concentrates, can be used to develop tests assessing the therapeutic potential of stromal cells intended for cellular therapy.

## Data Availability Statement

All datasets presented in this study are included in the article/supplementary material.

## Author Contributions

KM, MC, and NB contributed to the conception and design of the study. HL and NB carried out the majority of the experiments and statistical analyses. SM-C and YJ contributed to the experimental work. NB wrote the first draft of the manuscript. All authors contributed to manuscript revision and read and approved the submitted version.

## Conflict of Interest

The authors declare that there is intellectual property granted to KM and NB for the treatment of ARDS with ASC-CM and that Theratome Bio, Inc. has a business interest in this intellectual property.
